# Structural Neuroimaging Markers of Dementia: Insights from ROC Curve Analysis

**DOI:** 10.21203/rs.3.rs-7418658/v1

**Published:** 2025-09-17

**Authors:** Sam Pepper, Setorwu Dzesu, Lauren Yoksh, Ankit Patel, Robyn Honea, Dinesh Pal Mudaranthakam

**Affiliations:** University of Kansas Medical Center; University of Kansas Medical Center; University of Kansas Medical Center; University of Kansas, University of Kansas Medical Center; University of Kansas, University of Kansas Medical Center; University of Kansas Medical Center

**Keywords:** Neuroimaging, Dementia, Biomarkers, ROC Curves, Structural MRI

## Abstract

**Background:**

Alzheimer’s disease and related dementias (ADRD) are growing public health concerns. Early and accurate differentiation between cognitively normal (NL), mild cognitive impairment (MCI), and dementia is essential for timely intervention. Structural MRI biomarkers—such as grey matter volume (GMV), white matter volume (WMV), white matter hyperintensity volume (WMH), AD signature (a meta-region of interest), and hippocampal occupancy score (HOC)—offer promise for objectively staging cognitive decline.

**Objective:**

This study aimed to evaluate the diagnostic utility of MRI-derived neuroimaging measures using receiver operating characteristic (ROC) curve analysis to identify optimal thresholds for distinguishing between NL, MCI, and dementia.

**Methods:**

Data were collected from 466 participants at the KU Alzheimer’s Disease Research Center between 2010 and 2025. T1-weighted MRI scans were preprocessed using SPM12 and VBM12. Statistical analyses included ANOVA, Tukey’s HSD, Welch’s t-tests, and ROC analyses. Youden’s J statistic was used to determine optimal cut-points, and multivariate models assessed combined biomarker performance.

**Results:**

Significant differences were found across diagnostic groups for GMV, WMH, AD signature, and HOC. ROC analysis for Demented vs the control group (NL) showed GMV had an AUC of 0.674 [0.6164, 0.7325], WMH an AUC of 0.700 [0.6454, 0.7539], AD signature an AUC of 0.829 [0.780, 0.8761], and HOC the highest individual performance with an AUC of 0.878 [0.8426, 0.9144]. Further ROC analysis confirmed AD signature and HOC as the most effective biomarkers (AUCs = 0.78 and 0.81) for distinguishing MCI/demented from NL. In MCI vs. demented comparisons, AD signature and HOC biomarkers had AUCs of 0.70 and 0.73, respectively.

**Conclusions:**

MRI-derived biomarkers, particularly AD signature and HOC, show strong potential for differentiating cognitive impairment stages. ROC-based thresholds offer clinically actionable metrics that can improve diagnostic precision.

## Introduction

Alzheimer’s disease (AD) and related dementias (ADRD) continue to pose significant public health challenges, with rising prevalence and limited therapeutic options. Accurate and early differentiation of disease stages—particularly between cognitively normal (NL), mild cognitive impairment (MCI), and dementia—is critical for prognosis, timely intervention, and appropriate patient management (Jack et al., 2010; Sperling et al., 2011). Neuroimaging has emerged as a powerful tool in this effort, offering non-invasive biomarkers that reflect structural and pathological brain changes associated with ADRD.

In particular, structural MRI has provided insight into alterations in grey matter density and volume, white matter volume (WMV), and white matter hyperintensities (WMH), each of which is implicated in neurodegenerative and cerebrovascular processes contributing to cognitive decline (Frisoni et al., 2010; Du et al., 2001; Debette & Markus, 2010). Grey matter atrophy has been consistently associated with Alzheimer’s pathology, especially in the medial temporal lobe and neocortical regions (Fox et al., 2001–2007). Similarly, white matter degeneration and increased WMH burden are linked to small vessel disease and mixed dementia phenotypes, compounding the effects of primary neurodegeneration (Prins & Scheltens, 2015; Brickman et al., 2010).

Despite these advances, translating neuroimaging findings into clinically actionable tools remains a challenge. Current clinical practice often relies on qualitative interpretation or subjective thresholds, which may vary across institutions and practitioners (McEvoy & Brewer, 2010). By applying receiver operating characteristic (ROC) curve analyses to neuroimaging markers, we aim to derive optimal cut-points that can discriminate between NL, MCI, and dementia with high sensitivity and specificity (Youden, W. J. 1950). These data-driven thresholds can support clinical teams in making objective assessments and tracking disease progression with greater precision.

The KU Alzheimer’s Disease Center (KU ADC) offers a unique environment for investigating these relationships, featuring a well-characterized cohort, standardized imaging protocols, and structured diagnostic adjudication. Our work utilizes this rich dataset to evaluate the discriminative utility of MRI-derived measures of grey matter, white matter, and WMH in distinguishing stages of cognitive impairment. These efforts align with broader national initiatives to develop quantitative imaging biomarkers that enhance clinical decision-making and personalized care in Alzheimer’s disease-related dementia (ADRD) (Weiner et al., 2013; Hampel et al., 2021). The clinical utility of this approach is considerable. By providing threshold-based interpretations of neuroimaging findings, clinicians can more effectively distinguish between stages of cognitive impairment, particularly in diagnostically ambiguous cases. The longitudinal application of these metrics may aid in monitoring disease progression and identifying patients at high risk of converting to dementia. Moreover, standardized reporting of GMV, WMV, and WMH burden, tied to validated thresholds, can improve communication between radiologists, neurologists, primary care providers, and memory clinics. These findings also have implications for research infrastructure by enhancing recruitment strategies and precision in clinical trial design.

In summary, our study bridges the gap between neuroimaging science and clinical utility by offering statistically significant MRI-derived structural markers in the context of ADRD. By operationalizing brain health indicators into clinically meaningful thresholds, this work advances the vision of a learning health system capable of proactive dementia care.

## Methods

### Standard Protocol Approvals, Registrations, and Patient Consents:

Study procedures were approved by the University of Kansas School of Medicine Institutional Review Board and were in accordance with U.S. federal regulations. All participants provided written informed consent.

#### Data Source:

This analysis uses MRI brain scan data collected by the University of Kansas Alzheimer’s Disease Research Center (KU ADRC) from 2010 to 2025. The KU ADRC is part of the U.S. network of Alzheimer’s Disease Centers of Excellence that support research into brain aging and dementia. The KU ADRC has established an infrastructure for the identification, recruitment, and characterization of older adults both with and without dementia. Beginning in 2004, we developed a registry of individuals who have consented to be contacted regarding research studies, details of which have been published elsewhere (Vidoni et al., 2012). The KU ADRC obtains data on research participants as part of the National Alzheimer’s Coordinating Center (NACC) requirements. These data are obtained by research and ethical standards. Individuals whose data are extracted give consent for the use of data for research and clinical purposes. Participants were recruited as part of intervention and observational studies at the University of Kansas Alzheimer’s Disease Center (KU ADC) and were part of the Clinical Cohort. We have previously reported results from these investigations (Morris et al., 2017; Morris et al., 2020; Vidoni et al., 2015).

All participants also underwent a standard examination, which includes a thorough clinical and cognitive evaluation with a clinician at the KU ADC. This clinical evaluation includes a semi-structured interview (Clinical Dementia Rating, CDR) with the participant and study partner (Morris, 1993), as well as a physical and neurological examination. Clinical evaluation results were used to determine dementia status, which were reviewed along with psychometric battery results and finalized at a consensus diagnostic conference attended by clinicians and psychometricians using the NINCDS-ADRDA criteria as well as the McKann NIA-AA workgroup diagnostic guidelines (Beach et al., 2012; McKhann et al., 2011). Diagnostic criteria for AD require the gradual onset and progression of impairment in memory and in at least one other cognitive and functional domain on the CDR. MCI was diagnosed by a clinician and verified with medical records. Individuals were excluded from participating if they had other neurological disorders that could impair cognition, evidence of bleeding disorders during screening, clinically significant disease, psychiatric disorder, systemic illness, stroke, or myocardial infarction.

The following categories classified the cognition status of participants in the cohort; Mild Cognitive Impairment (MCI), Impaired, Demented (including AD) and the Control or healthy group (NL). Individuals who were cognitively unimpaired (CU) were included at age 60 and older, while individuals with a dementia diagnosis were included regardless of age. The Uniform Data Set (UDS) was created in 2005 to collect standard clinical data on participants from the National Institute on Aging (NIA)-supported Alzheimer’s Disease Centers (ADCs). The UDS is administered to ADC Clinical Cohort participants on an approximately annual basis.

The data obtained include participant cognition status, demographic information and other health related indicators like systolic and diastolic blood pressure, heart rate, sleep apnea, BMI, cardiac arrest, arthritis, incontinence, seizures, diabetes status, insomnia, alcohol intake, ptsd, bipolar, anxiety, vision, hearing, hypertension, hypercholesterolemia etc.

#### Preprocessing of MRI Data:

**Figure 1:**
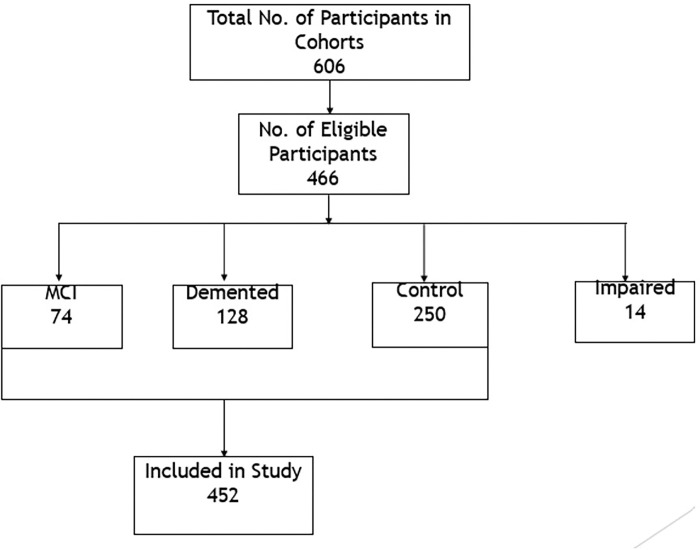
Analytical Data Set

All participants coming through neuroimaging studies at the KUADC underwent magnetic resonance imaging (MRI) of the brain in either a Siemens 3.0 Tesla Allegra or Skyra scanner. We obtained a high resolution T1-weighted image (MP-RAGE; 1×1×1mm voxels; TR = 2500ms, TE = 4.38ms, TI = 1100, FOV = 256×256 with 18% oversample, 1mm slice thickness, flip angle 8deg) for detailed anatomy with high gray-white matter contrast. Every scan was checked for image artifacts and gross anatomical abnormalities. 466 individuals with MPRAGE scans passed quality control. For volumetric pre-processing of T1-weighted images, we used the Computational Anatomical Toolbox 12 (CAT12 Version 12.6, C. Gaser, Structural Brain Mapping Group, Jena University Hospital, Jena, Germany; http://dbm.neuro.uni-jena.de/cat/) through Statistical Parametric Mapping version 12 (SPM12; Wellcome Trust Centre for Neuroimaging, London, UK; http://www.fil.ion.ucl.ac.uk/spm/software/spm12/)) that operate under Matlab (R2023a) (the Mathworks, Natick, MA) on Mac. T1 images were corrected for bias-field inhomogeneities, registered using linear (12-parameter affine) and non-linear transformations, spatially normalized using the high-dimensional DARTEL algorithm into MNI space(Ashburner, 2007), and segmented into gray matter (GM), white matter (WM), cerebrospinal fluid (CSF) and white matter hyperintensity (WMH). We calculated total intracranial volume (TIV) using total gray, white, and CSF volumes. We chose to use two specific calculated imaging biomarkers that are sensitive to disease progression and AD genetic risk [30, 31]. We computed a hippocampal occupancy score (HOC) as an estimate of medial temporal lobe atrophy, by determining the ratio of left and right hippocampal volume to the sum of the hippocampal and interior lateral ventricular volumes. HOC has been shown to have a higher discriminative accuracy than the standard hippocampal volume measure [32]. We also calculated an AD Signature composite which included gray matter medial (hippocampus, parahippocampus, entorhinal cortices), middle and inferior and superior temporal cortex volumes (regions summarized in Fig. 1, hippocampus not shown) [33, 34].

#### Data Processing:

Box plots visualizing the distribution of grey matter volume, white matter volume, white matter hyperintensities, AD signature, and hippocampal occupancy score (HOC) were constructed based on the four primary diagnosis categories and summary statistics were observed. Analysis of variance (ANOVA) tests were performed to test for differences in distribution between primary diagnosis groups for these five variables of interest. Tukey’s Honest Significant Difference (HSD) tests were evaluated following the ANOVA tests to detect significant differences between specific primary diagnosis groups, and confidence intervals for specific group comparisons were constructed. Density plots were created to compare the densities of the distributions of the five variables of interest for primary diagnosis groups Demented, MCI, and NL.

Similar analyses were performed comparing only demented and NL primary diagnosis groups. Welch two-sample t-tests were performed to compare the means of the two groups for each of the five variables of interest.

#### ROC Curve:

Using the collapsed version of the data set in which primary diagnosis has two values, Demented and NL. We also considered the scenarios of MCI vs the control group (NL). We further combined the MCI group with the Demented group against the control. ROC curves were constructed for grey matter, white matter, white matter hyperintensities, AD signature, and HOC. The ROC curves were produced using the roc function for each scenario. This data was then plotted to create the ROC curves, with sensitivity on the y-axis and 1-specificity on the x-axis. Youden’s J statistics were calculated to determine the optimal cut-point for each ROC curve, and this point was added to the ROC curve plots (Youden, W. J. 1950). The area under the curve (AUC) together with 95% CIs were calculated for each ROC curve and reported on the plots. In the scenarios of MCI and Demented vs control, and MCI vs control, ROC curves were illustrated in a single plot for better visualization of biomarker performance. All analyses were performed using R version 4.5.0.

## Results

The final cohort for this study includes a total of 466 participants. The primary diagnoses of the cohort include 128 dementia, 14 impaired, 74 mild cognitive impairment (MCI), and 250 cognitively normal (NL). No significant difference in age or race was found between MCI and demented groups, nor between NL and the combined MCI and demented groups. There was a significant difference in years of education between MCI and Demented groups as well as between NL and combined MCI and demented groups.

ANOVA tests found significant differences in the distributions of grey matter volume, white matter hyperintensities, AD signature, and HOC between the four primary diagnosis groups. Significant differences in grey matter were found between diagnosis groups impaired versus demented, MCI versus demented, and NL versus demented. Welch’s two-sample t-test for demented and NL diagnosis groups found a significant difference in grey matter distribution between the two groups (p < 0.00001). Among the demented and NL diagnosis groups, the sensitivity for the grey matter model was found to be 0.7028 with a specificity of 0.5859 and an AUC of 0.674. The optimal threshold (Youden’s J) for grey matter was 571.60.

Significant differences in white matter hyperintensities were found between diagnosis groups NL versus demented and NL versus impaired, and the two-sample t-test provided a significant result comparing demented versus NL groups (p < 0.00001). Comparing demented versus NL groups, the white matter hyperintensities model had a sensitivity of 0.5221 with a specificity of 0.7734 and an AUC of 0.700. The optimal threshold for white matter hyperintensities was 3.14.

Significant differences in AD signature were found between diagnosis groups impaired versus demented, MCI versus demented, NL versus demented, MCI versus impaired, and NL versus MCI. The two-sample t-test comparing the collapsed two-diagnosis group data set found a significant difference in AD signature between demented and NL groups (p < 0.00001). The AD signature model had a sensitivity of 0.8835 with a specificity of 0.6797 and an AUC of 0.829. The optimal threshold for AD signature was determined to be 0.1084.

Significant differences in HOC were found between diagnosis groups impaired versus demented, MCI versus demented, NL versus demented, and NL versus MCI. The two-sample t-test provided a significant result comparing demented versus NL groups (p < 0.00001). The sensitivity for the HOC model was found to be 0.7831 with a specificity of 0.8047 and an AUC of 0.878. The optimal threshold for HOC was determined to be 0.9695.

No significant difference was found for the distribution of white matter between the four diagnosis groups. Comparing demented versus NL groups, the sensitivity for the white matter model was found to be 0.7229 with a specificity of 0.4219 and an AUC of 0.571. The optimal threshold for white matter was 433.21.

The model comparing the combined diagnosis group MCI and demented with NL using all five predictor variables of interest provided similar results to the univariate models, with AD signature and HOC providing the largest AUC out of the five ROC curves (AUC = 0.78 and 0.81, respectively). The optimal thresholds for AD signature (0.1085) and HOC (0.9736) obtained from this model were nearly identical to those obtained from the univariate models. The resulting thresholds for grey matter volume, white matter volume, and white matter hyperintensities were 572.59, 409.55, and 2.42, respectively, which are similar to the thresholds reported from the univariate models. From this model, AD signature has the highest specificity at 0.8795, and white matter hyperintensities has the lowest specificity of 0.3815. White matter hyperintensities, however, has the highest sensitivity in this model, which was found to be 0.6884, and a corresponding specificity of 0.8366, and white matter volume has the lowest sensitivity of 0.2277. AD signature and HOC have the overall best operating characteristics in this model.

The model comparing MCI versus demented diagnosis groups as a function of all five variables did not provide as strong of results as the previous model. Once again, the ROC curves for AD signature and HOC provide the largest AUC, 0.70 and 0.73 respectively, and have similar thresholds to those found from previous models (0.1055 and 0.9607, respectively). In this model, white matter hyperintensity has the highest specificity of 0.7656, but the lowest corresponding sensitivity at 0.4865. This model resulted in a specificity of 0.5 and a sensitivity of 0.8649 for AD signature. HOC has a specificity of 0.6953 and sensitivity of 0.6622.

## Discussion

This study demonstrates the potential of structural MRI-derived biomarkers—grey matter density, grey matter volume (GMV), white matter volume (WMV), and white matter hyperintensities (WMH)—to differentiate clinical stages of cognitive impairment along the Alzheimer’s disease continuum. By applying ROC curve analysis to a rigorously phenotype cohort from the KU Alzheimer’s Disease Center, we quantified the discriminative performance of these imaging features in distinguishing between normal cognition (NL), mild cognitive impairment (MCI), and dementia. Our results support the hypothesis that neuroimaging metrics can serve not only as correlates of cognitive status but also as diagnostic tools when linked to clinically relevant thresholds.

Among the imaging features assessed, grey matter volume and WMH burden showed strong classification performance, particularly in differentiating Demented from MCI and NL participants. This aligns with previous research demonstrating that cortical atrophy and cerebrovascular pathology are key hallmarks of Alzheimer’s disease and related dementias (Jack et al., 2010; Brickman et al., 2012; Prins & Scheltens, 2015). The use of empirically derived thresholds via ROC optimization offers practical advancement: instead of relying solely on z-scores or percentiles, clinicians can now consider actionable cut-off points in imaging interpretation to support diagnostic clarity (Falahati et al., 2014).

One of the most notable contributions of this work is the generation of threshold values that may be translated into clinical workflows. For example, radiologists and neurologists could integrate these findings into structured reporting formats, highlighting when GMV or WMH burden exceeds thresholds predictive of MCI or dementia. This supports a more standardized and objective approach to neuroimaging interpretation, reducing variability and facilitating interdisciplinary decision-making (Weiner et al., 2017). Moreover, by incorporating these metrics into clinical dashboards or EHR-integrated decision support systems, longitudinal tracking of brain health can become more feasible within routine practice (Tosun et al., 2016).

From a research infrastructure perspective, the integration of ROC-based thresholds enhances precision recruitment for clinical trials. Individuals who meet imaging criteria for early neurodegeneration may be prioritized for enrollment in prevention studies, thereby improving cohort enrichment strategies (Donohue et al., 2014). Similarly, these tools can aid in the stratification of participants in observational studies based on neuroimaging severity, leading to more robust subgroup analyses and outcome modeling (Dyrba et al., 2015).

However, this study is not without limitations. First, the diagnostic categories used as reference standards are based on clinical consensus, which may be subject to interrater variability (Albert et al., 2011). Second, while our sample reflects the characteristics of a tertiary Alzheimer’s Disease Center, generalizability to more diverse or community-based populations remains to be tested (Morris et al., 2006). Future work should examine how these thresholds perform across different MRI acquisition protocols and scanner types to ensure broader applicability. Additionally, combining structural imaging with other modalities—such as functional MRI, PET imaging, or blood-based biomarkers—may enhance predictive accuracy and support multidimensional diagnostic models (Hampel et al., 2018).

In conclusion, this work represents a critical step toward operationalizing brain health metrics for clinical and translational purposes. By deriving and validating imaging thresholds for key neuroanatomical features, we move closer to a precision medicine approach to dementia care—one that supports early identification, individualized monitoring, and timely intervention. These findings lay the groundwork for future integration into digital health platforms, automated reporting tools, and adaptive learning systems, ultimately enhancing care for individuals across the Alzheimer’s disease spectrum.

## Figures and Tables

**Figure 2:**
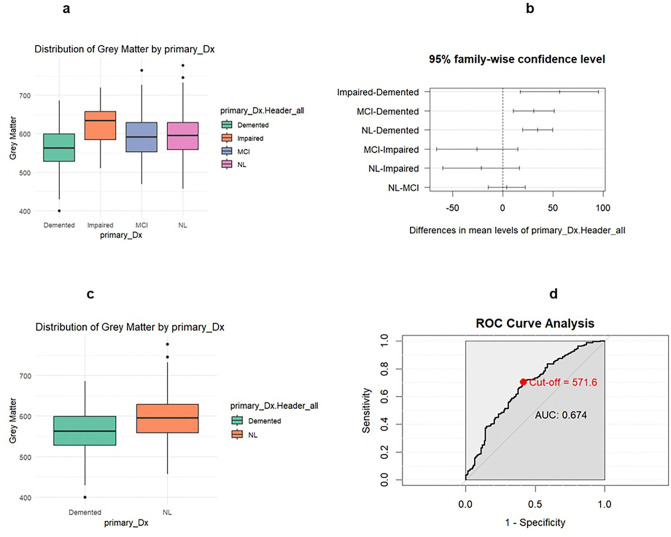
Legend not included with this version

**Figure 3:**
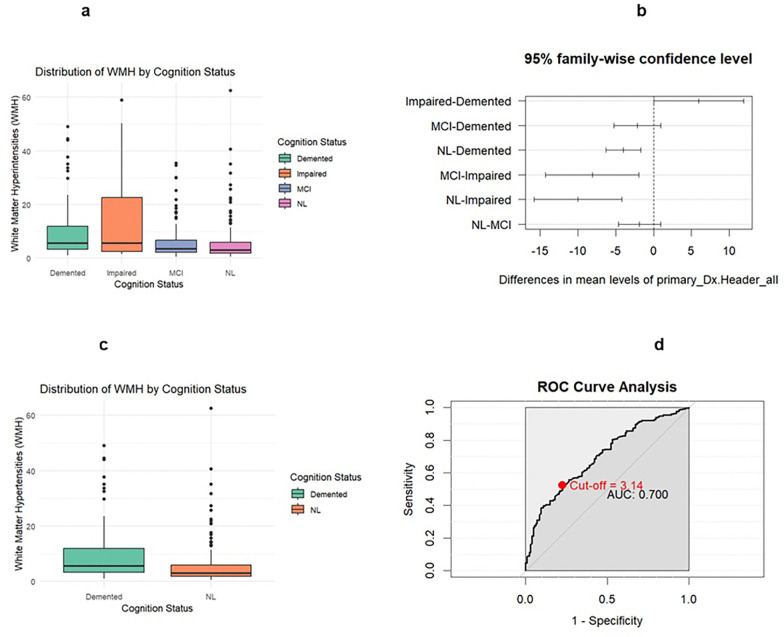
Legend not included with this version

**Figure 4:**
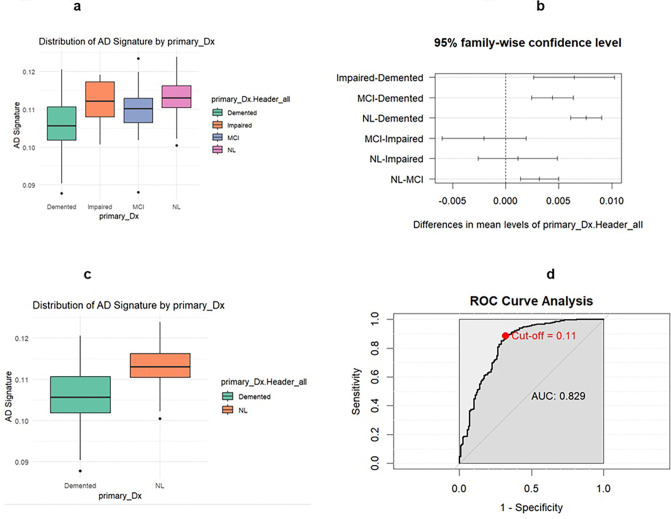
Legend not included with this version

**Figure 5:**
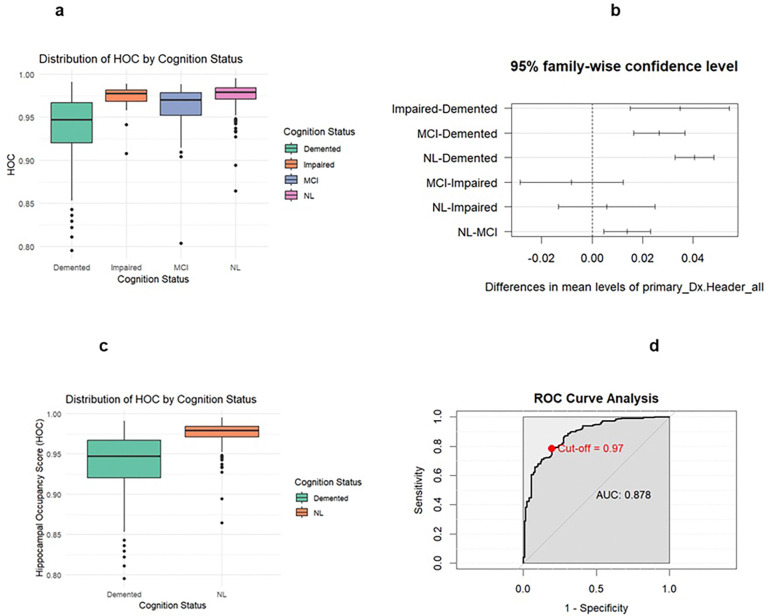
Legend not included with this version

**Figure 6:**
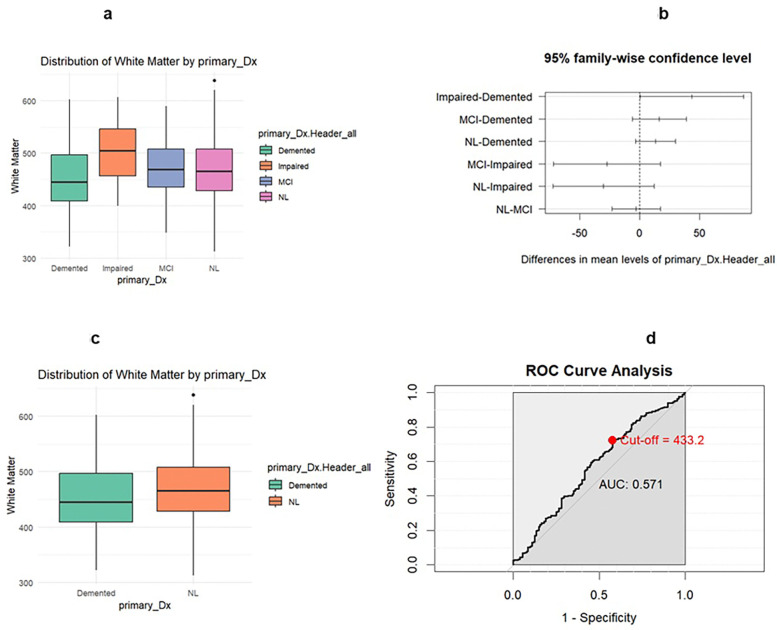
Legend not included with this version

**Figure 7:**
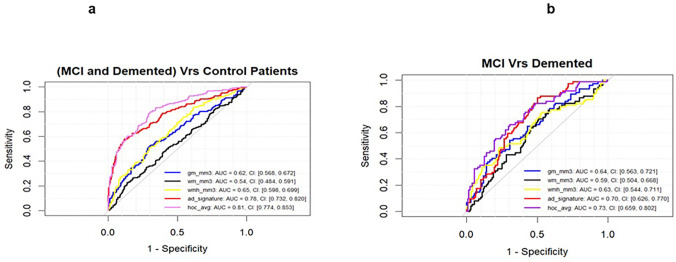
Legend not included with this version

**Table 1:** 

Variable/Biomarker	MCI & Demented (n = 202)	Control (n = 250)	P-value	MCI (n = 137)	Demented (n = 216)	P-value
Age at Visit Mean(sd)	74.8 (7.71)	73.8 (6.56)	0.1638	73.9 (7.52)	75.2 (7.81)	0.2392
Presumed Disease Status at Enrollment			**< 0.0001**			**0.0346**
Case	38 (0.86)	6 (0.14)		7(0.18)	31(0.82)	
Control	8 (0.16)	42 (0.84)		3(0.38)	5(0.62)	
No Presumed Disease	156 (0.44)	202 (0.56)		64 (0.41)	92 (0.59)	
Sex			**< 0.0001**			0.7032
Male	107 (0.56)	85 (0.44)		41(0.38)	66(0.62)	
Female	95 (0.37)	165 (0.63)		33(0.35)	62(0.65)	
Race			0.6792			0.7633
White	185 (0.45)	230 (0.55)		67 (0.36)	118 (0.64)	
Black or African American	12 (0.43)	16 (0.57)		5 (0.42)	7 (0.58)	
American Indian or Alaska Native	0 (0.00)	1 (1.00)		0	0	
Asian	1 (0.33)	2 (0.67)		0 (0.00)	1 (1.00)	
Others	4 (0.80)	1 (0.20)		2 (0.50)	2 (0.50)	
Education (Number of years)	15.7 (3.10)	16.7 (2.58)	**0.0003**	16.1 (3.26)	15.5 (2.99)	0.1806
Mean(sd)						
Gray Matter	573.01 (58.57)	596.35 (50.93)	**< 0.0001**	592.46 (58.25)	561.76 (55.95)	**0.0003**
White Matter Volume	460.87 (60.00)	468.18 (59.16)	0.1961	471.23 (58.55)	454.88 (60.23)	0.0603
White Matter Hyperintensities	8.37 (8.91)	5.14 (6.59)	**< 0.0001**	7.00 (8.23)	9.15 (9.22)	0.0888
AD Signature	0.1073 (0.01)	0.1132 (0.00)	**< 0.0001**	0.1100 (0.01)	0.1056 (0.01)	**< 0.0001**
HOC	0.9444 (0.04)	0.9753 (0.02)	**< 0.0001**	0.9613 (0.02)	0.9346 (0.04)	**< 0.0001**

ANOVA tests found significant differences in the distributions of grey matter volume, white matter hyperintensities, AD signature, and HOC between the four primary diagnosis groups. Significant differences in grey matter were found between diagnosis groups impaired

**Table 2 a:** Four diagnosis group Grey Matter summary statistics

Cognition Status	Count	Mean	Median	SD	IQR	Q1	Q3	Min	Max
Demented	128	561.76	562.60	55.95	70.86	528.31	599.16	399.57	686.09
Impaired	14	618.12	633.70	63.86	73.21	584.28	657.48	510.31	720.02
MCI	74	592.46	591.25	58.25	76.51	552.54	629.05	469.35	764.16
NL	250	596.35	595.93	50.93	69.49	559.17	628.66	456.95	776.86

**Table 2 b:** Two diagnosis group Grey Matter summary statistics

Cognition Status	Count	Mean	Median	SD	IQR	Q1	Q3	Min	Max
Demented	128	561.76	562.60	55.95	70.86	528.31	599.16	399.57	686.09
NL	250	596.35	595.93	50.93	69.49	559.17	628.66	456.95	776.86

**Table 3 a:** Four diagnosis group White Matter Hyperintensities summary statistics

Cognition Status	Count	Mean	Median	SD	IQR	Q1	Q3	Min	Max
Demented	128	9.1537	5.570	9.218	8.620	3.265	11.885	0.99	49.082
Impaired	14	15.120	5.570	18.73	20.123	2.525	22.652	1.53	58.860
MCI	74	7.003	3.445	8.228	4.523	2.153	6.675	0.58	35.345
NL	250	5.134	2.960	6.595	4.070	1.880	5.950	0.50	62.420

**Table 3 b:** Two diagnosis group White Matter Hyperintensities summary statistics

Cognition Status	Count	Mean	Median	SD	IQR	Q1	Q3	Min	Max
Demented	128	9.1537	5.570	9.218	8.620	3.265	11.885	0.99	49.082
NL	250	5.134	2.960	6.595	4.070	1.880	5.950	0.50	62.420

**Table 4 a:** Four diagnosis group AD Signature summary statistics

Cognition Status	Count	Mean	Median	SD	IQR	Q1	Q3	Min	Max
Demented	128	0.1056	0.1056	0.007	0.009	0.1019	0.1106	0.0878	0.1205
Impaired	14	0.1121	0.1122	0.006	0.009	0.1080	0.1173	0.1007	0.1191
MCI	74	0.1100	0.1102	0.005	0.006	0.1065	0.1130	0.0880	0.1234
NL	250	0.1132	0.1130	0.004	0.006	0.1105	0.1162	0.1004	0.1238

**Table 4 b:** Two diagnosis group AD Signature summary statistics

Cognition Status	Count	Mean	Median	SD	IQR	Q1	Q3	Min	Max
Demented	128	0.1056	0.1056	0.007	0.009	0.1019	0.1106	0.0878	0.1205
NL	250	0.1132	0.1130	0.004	0.006	0.1105	0.1162	0.1004	0.1238

**Table 5 a:** Four diagnosis group HOC summary statistics

Cognition Status	Count	Mean	Median	SD	IQR	Q1	Q3	Min	Max
Demented	128	0.935	0.947	0.042	0.047	0.920	0.967	0.795	0.991
Impaired	14	0.970	0.977	0.022	0.013	0.969	0.981	0.908	0.989
MCI	74	0.961	0.970	0.028	0.027	0.952	0.979	0.804	0.989
NL	250	0.976	0.979	0.015	0.013	0.971	0.985	0.864	0.995

**Table 5 b:** Two diagnosis group HOC summary statistics

Cognition Status	Count	Mean	Median	SD	IQR	Q1	Q3	Min	Max
Demented	128	0.935	0.947	0.042	0.047	0.920	0.967	0.795	0.991
NL	250	0.976	0.979	0.015	0.013	0.971	0.985	0.864	0.995

**Table 6 a:** Four diagnosis group White Matter summary statistics

Primary Diagnosis	Count	Mean	Median	SD	IQR	Q1	Q3	Min	Max
Demented	128	454.88	445.00	60.23	87.33	409.29	496.63	321.65	602.43
Impaired	14	498.38	504.77	61.10	90.05	456.58	546.61	399.59	606.40
MCI	74	471.23	468.47	58.55	72.72	435.45	508.17	348.77	589.40
NL	250	468.18	464.96	59.16	79.03	428.55	507.58	312.58	637.87

**Table 6 b:** Two diagnosis group White Matter summary statistics

Group	Count	Mean	Median	SD	IQR	Q1	Q3	Min	Max
Demented	128	454.88	445.00	60.23	87.33	409.29	496.63	321.65	602.43
NL	250	468.18	464.96	59.16	79.03	428.55	507.58	312.58	637.87

**Table 7:** 

Biomarker	MCI&Demented vs Control			MCI vs Demented		
	Threshold	Specificity	Sensitivity	Threshold	Specificity	Sensitivity
Gray Matter	572.59	0.6988	0.5248	587.545	0.6953	0.5405
White Matter Volume	409.455	0.8635	0.2277	442.265	0.4922	0.7027
White Matter Hyperintensities	2.42	0.3815	0.8366	3.17	0.7656	0.4865
AD Signature	0.1085	0.8795	0.5792	0.1055	0.5	0.8649
HOC	0.9736	0.7068	0.7871	0.9607	0.6953	0.6622

## Data Availability

Detailed data cannot be shared publicly to protect the privacy of individual participants. Information to support the findings of these analyses is available by contacting the corresponding author, who, upon reasonable request and understanding of the intended use of the data, will provide the requested information in a manner that continues to protect individual patient information.
